# Cognitive Performance in Transfusion-Dependent Adults with β-Thalassemia in Bulgaria: A Case–Control Study

**DOI:** 10.3390/neurolint18060101

**Published:** 2026-05-22

**Authors:** Viktoria Babacheva, Kostadin Kostadinov, Veselina Goranova-Marinova, Miroslava Hristova, Penka Atanassova

**Affiliations:** 1 Department of Hematology, Medical University of Plovdiv, 4002 Plovdiv, Bulgaria; veselina.goranova@mu-plovdiv.bg; 2 Clinic of Hematology, University Hospital for Active Treatment “Sv. Georgi” (UMHAT “Sv. Georgi”), 4000 Plovdiv, Bulgaria; 3 Department of Social Medicine and Public Health, Medical University of Plovdiv, 4002 Plovdiv, Bulgaria; kostadinr.kostadinov@mu-plovdiv.bg; 4 Department of Neurology, Medical University of Plovdiv, 4002 Plovdiv, Bulgaria; miroslava.hristova@mu-plovdiv.bg (M.H.); penka.atanasova@mu-plovdiv.bg (P.A.); 5 Clinic of Neurology, University Hospital for Active Treatment “Sv. Georgi” (UMHAT “Sv. Georgi”), 4000 Plovdiv, Bulgaria

**Keywords:** β-thalassemia, cognitive impairment, MoCA, executive function, Trail Making Test, case–control study, adults, Bulgaria

## Abstract

**Background**: As survival improves in transfusion-dependent β-thalassemia, long-term adult morbidity, including cognitive dysfunction, has become increasingly relevant. Adult data remain limited, particularly in Eastern Europe, and many studies rely on single screening tools with limited control for confounding. **Methods**: We conducted a single-center case–control study (2024–2025) at the Congenital Hemolytic Anemia Treatment Center, University Hospital “Sv. Georgi” Plovdiv, Bulgaria. Fifty adults with transfusion-dependent β-thalassemia (86% thalassemia major; 14% transfusion-dependent intermedia) and 30 frequency-matched healthy controls completed a multi-domain cognitive battery: Montreal Cognitive Assessment (MoCA), Mini-Mental State Examination (MMSE), Clock Drawing Test (CDT), Trail Making Test (TMT-A/B), and timed verbal fluency. Associations between thalassemia status and cognitive outcomes were estimated using three prespecified models: unadjusted, adjusted for age and sex, and a doubly robust model combining covariate balancing propensity score inverse probability weighting (balancing BMI, smoking, education, and comorbidity) with age/sex regression adjustment. **Results**: Patients performed worse than controls on global cognition and executive/visuospatial measures. MoCA scores were lower in patients (−2.26 unadjusted, *p* = 0.016; −2.83 doubly robust, *p* = 0.001), as were MMSE scores (−1.64, *p* = 0.015; −1.87, *p* = 0.002). CDT performance was consistently poorer (OR ≈ 0.28–0.30 across models). Patients were slower on TMT-B (time ratio 1.35 unadjusted, *p* = 0.003; 1.42 doubly robust, *p* < 0.001); TMT-A reached significance only after weighting (ratio 1.32, *p* = 0.001). Verbal fluency was modestly lower with borderline significance (*p* ≈ 0.05–0.06). **Conclusions**: Transfusion-dependent β-thalassemia in adults is associated with poorer cognitive performance, particularly in global cognition and executive/visuospatial domains, with results robust across adjustment strategies. Routine multi-domain cognitive screening may be warranted in adult thalassemia care.

## 1. Introduction

Thalassemias are inherited hemoglobin disorders transmitted in an autosomal recessive manner and represent one of the most prevalent congenital hemolytic anemias worldwide [1,2]. Their global distribution spans a broad geographic belt including the Mediterranean region, the Middle East, Central and Southeast Asia, India, South China, and parts of Africa and South America [3]. Within this belt, epidemiological patterns vary considerably, reflecting historical migration, carrier frequency, and healthcare organization.

Bulgaria occupies an intermediate position within this distribution. The patient population is concentrated predominantly in the southern regions and managed through a limited number of specialized tertiary centers, creating a centralized care within a healthcare context that remains underrepresented in the international literature [4,5,6]. National epidemiological data, however, are limited and not systematically updated. Registry data indicate 270 patients in 2012, of whom 92.2% had thalassemia major and 7.8% had transfusion-dependent thalassemia intermedia [4], increasing to 287 patients in 2015, including 250 with thalassemia major [5]. Although numerically modest, this represents a stable population receiving lifelong specialized care.

At the molecular level, β-thalassemia results from pathogenic variants in the HBB gene on chromosome 11, leading to reduced (β^+^) or absent (β^0^) β-globin synthesis [7]. The resulting imbalance between α- and β-globin chains leads to ineffective erythropoiesis, chronic hemolysis, and compensatory extramedullary hematopoiesis. Clinically severe forms are characterized by chronic anemia, organomegaly, and progressive iron overload due to both repeated transfusions and increased intestinal iron absorption [8]. Advances in transfusion and iron chelation therapy have markedly improved survival, transforming β-thalassemia from a fatal pediatric condition into a chronic adult disease requiring lifelong multidisciplinary management [9,10].

Consequently, as survival has improved, clinical attention has progressively shifted from mortality to long-term morbidity and quality of life. While somatic complications, including cardiovascular, hepatic, skeletal, and endocrine involvement, are well-characterized [11,12,13], neuropsychological outcomes remain comparatively underexplored. Lower cognitive performance has been reported in both pediatric and adult patients, particularly affecting executive function, attention, and processing speed [10,14,15,16,17], with prevalence estimates ranging from 20% to 60% [18,19]. However, existing evidence is predominantly derived from pediatric or mixed-age cohorts, where developmental factors and cumulative disease burden differ substantially from the adult context [20,21]. This distinction is clinically relevant, as cognitive impairment in adults directly affects occupational capacity, independence, and social participation.

However, methodological limitations persist. Many studies rely on intelligence testing or a single global screening tool, limiting clinical applicability and domain-specific assessment. In contrast, multi-domain instruments suitable for routine clinical use offer greater practical relevance. The Montreal Cognitive Assessment has demonstrated superior sensitivity for mild cognitive impairment compared to the Mini-Mental State Examination, particularly in executive and visuospatial domains [22,23], while complementary tools such as the Clock Drawing Test and Trail Making Test capture visuospatial ability, processing speed, and cognitive flexibility [24,25,26].

Despite these advances, important gaps remain. The multidomain cognitive profile of transfusion-dependent adults has not been systematically characterized. Studies frequently rely on single instruments, and adjustment for confounding is often limited. In addition, data from Eastern European populations, including Bulgaria, remain scarce.

The present study aimed to evaluate cognitive performance in adults with transfusion-dependent β-thalassemia and to compare outcomes with those of a frequency-matched healthy control group. Primary endpoints included global cognition assessed by the Montreal Cognitive Assessment (MoCA) and visuospatial–executive function measured by the Clock Drawing Test. Secondary endpoints comprised Mini-Mental State Examination performance, Trail Making Test parts A and B, and verbal fluency.

## 2. Materials and Methods

### 2.1. Study Design and Participants

A single-center case–control study was conducted between 2024 and 2025 at the Congenital Hemolytic Anemia Treatment Center, Clinic of Clinical Hematology, University Hospital “St. George”, Plovdiv, Bulgaria. This center serves as a regional referral unit for adult patients with transfusion-dependent β-thalassemia.

The case group comprised 50 adult patients (≥18 years) with transfusion-dependent β-thalassemia who were actively followed at the center. Of these, 43 (86%) had thalassemia major and 7 (14%) had transfusion-dependent thalassemia intermedia. Given the rarity of the disease and the exhaustive recruitment of all eligible patients attending the center during the study period, a formal a priori sample size calculation was not performed. The case group size was determined by the total available population rather than by a pre-specified target. As a result, the modest sample size may limit the statistical power to detect small or moderate differences in cognitive outcomes, and some subgroup analyses may be underpowered or not feasible, which is addressed in Section 4.5.

Exclusion criteria for cases were concurrent acute illness, recent hospitalization for a non-thalassemia condition, known neurological disease unrelated to thalassemia, or inability to provide informed consent.

Controls were recruited from individuals attending the hospital for routine health check-ups or minor, self-limited conditions not associated with chronic systemic or neurological disease. All controls underwent structured clinical screening, review of medical records, and laboratory testing to exclude major medical conditions that could affect cognitive performance.

Controls were recruited with the intention of group-level frequency matching by age and sex to enhance comparability at the design stage. However, because individual matching identifiers were not retained and the case–control ratio varied across strata, analyses were conducted using an unmatched framework with covariate adjustment. Accordingly, matching was treated as a design feature rather than an analytical constraint, and confounding was addressed using regression and propensity score-based methods.

Control participants were required to be ≥18 years of age and free of known hemoglobinopathy, major systemic illness, or neurological disorder. Screening was conducted in all controls using a structured clinical interview, review of available medical records, and measurement of full blood count at enrollment.

The study was conducted in accordance with the ethical principles of the Declaration of Helsinki and Good Clinical Practice and complied with applicable Bulgarian laws and regulations governing clinical and scientific research involving human participants. All participants were interviewed prior to enrollment, and the study objectives and procedures were thoroughly explained. Written informed consent was obtained from all participants. Ethical approval was granted by the Local Ethics Committee at the Medical University of Plovdiv (protocol No. P-KHE-15/31 December 2024).

### 2.2. Demographic and Clinical Data Collection

Demographic data (date of birth, sex, marital status, and education) were obtained via structured interview. Anthropometric measurements (height in centimeters and weight in kilograms) were performed under standardized conditions, and body mass index (BMI, kg/m^2^) was calculated.

Clinical history included smoking status, prior thrombotic events, splenectomy, physician-diagnosed depression, and comorbid conditions. Psychosocial self-reported variables, including school absence due to illness, perception of overprotective parenting, and childhood feelings of inferiority, were assessed using a structured questionnaire.

For patients, disease-specific variables were recorded, including thalassemia subtype, year of diagnosis, chelation therapy, chelator type, and annual transfusion requirement expressed both as milliliters and units per year.

### 2.3. Hematological and Serological Assessment

Venous blood samples were collected at enrollment under standardized conditions and analyzed within four hours of collection. The timing was consistent across all patients and reflects the standard follow-up protocol of the center. Hemoglobin concentration measured at this visit represents the pre-transfusion baseline value, reflecting underlying disease severity. Cognitive assessments were conducted 48–72 h post-transfusion under standardized conditions, ensuring hemodynamic stability and minimizing the acute effects of anemia.

A complete blood count was performed using a calibrated automated hematology analyzer, including hemoglobin (g/L), red blood cell count (T/L), hematocrit (fraction), white blood cell count (G/L), platelet count (G/L), mean corpuscular volume (fL), mean corpuscular hemoglobin (pg), and mean corpuscular hemoglobin concentration (g/L). Serum ferritin (ng/mL) was measured by immunoassay as a marker of systemic iron burden. Aspartate aminotransferase (AST) and alanine aminotransferase (ALT) activities (U/L) were measured using standard enzymatic assays to evaluate hepatocellular function. Hepatitis B surface antigen (HBsAg) and hepatitis C antibody (anti-HCV) status were determined by validated immunoassay methods.

### 2.4. Cognitive Assessment

Cognitive function was evaluated using a standardized neurocognitive battery administered by trained investigators in a quiet clinical setting. Investigators were trained in standardized administration and scoring procedures prior to study initiation. Investigators were not blinded to participant group status, which represents a potential source of observer bias and is addressed in the Section 4.5. All cognitive testing was performed under standardized clinical conditions 48–72 h after routine transfusion, once the acute post-transfusion hemodynamic response had stabilized.

The Montreal Cognitive Assessment (MoCA) served as the principal screening instrument (range 0–30), with subscale scores recorded for visuospatial–executive function, naming, attention, language, abstraction, delayed recall, and orientation [27]. A total score of ≥26 was considered normal, 18–25 indicated mild impairment, and <18 indicated more severe impairment. The total score was incremented by one point for participants with less than 12 years of formal education. The Mini-Mental State Examination (MMSE) was administered concurrently as a comparator measure of global cognition. The MMSE comprises 11 items covering orientation to time and place, registration, attention and calculation, delayed recall, language, and visuoconstruction, resulting in a total score ranging from 0 to 30. Scores of 24 or above were considered indicative of intact global cognition, scores of 18 to 23 of mild-to-moderate impairment, and scores below 18 of more severe impairment [28].

Visuospatial–executive abilities were additionally assessed using the Clock Drawing Test (CDT). Participants were instructed to draw a clock face marking all 12 h and to set the hands to ten past eleven. Performance was scored using the Shulman five-point ordinal system, where scores of 4 or 5 indicate normal visuospatial–executive function and scores of 3 or below indicate impairment [29,30].

Processing speed and cognitive flexibility were evaluated using the Trail Making Test (TMT), Parts A and B. In Part A, participants connected 25 numbered circles in ascending order as quickly as possible, assessing visuomotor scanning and basic processing speed. In Part B, participants alternated between numbers and letters in ascending sequence (1–A–2–B, continuing to 13), imposing an additional set-shifting and executive control demand. Completion time in seconds was recorded for each part; shorter times indicate better performance. The TMT B/A ratio was computed as an index of executive processing load relative to basic visuomotor speed [31].

Semantic verbal fluency was assessed by instructing participants to generate as many words as possible belonging to a specified category within 60 s. The total number of correct, non-repeated responses produced within the time limit was recorded as a continuous measure [32].

All cognitive assessments were conducted in Bulgarian using validated versions of the instruments. Participants unable to complete a specific test were treated as missing for that outcome.

### 2.5. Statistical Analysis

Prior to analysis, data were screened for implausible values; laboratory measurements outside clinically plausible ranges were treated as missing. All models were estimated using complete cases for each outcome.

Continuous variables are presented as mean ± standard deviation, and categorical variables as counts and percentages. Baseline between-group differences were assessed using independent-samples *t*-tests with Welch correction and Pearson χ^2^ or Fisher’s exact tests as appropriate.

The prespecified primary outcomes were the MoCA total score and the CDT score. Secondary outcomes were MMSE total score, TMT-A and TMT-B completion times, TMT B/A ratio, and verbal fluency score. Participants unable to complete TMT-A or TMT-B were assigned worst-performance thresholds (90 and 273 s, respectively) [33] to reduce informative missingness. The TMT B/A ratio was then calculated.

Outcomes were modeled according to their distributional properties. MoCA, MMSE, and verbal fluency were analyzed using linear regression on the raw scale; therefore, the presented coefficients represent mean score differences. TMT-A, TMT-B, and the B/A ratio are right-skewed and were log-transformed prior to linear regression. Exponentiated coefficients from the log models are reported as multiplicative time ratios. The CDT was analyzed using proportional odds logistic regression; coefficients are reported as odds ratios for belonging to a higher performance category, with OR < 1 indicating lower odds of higher performance in patients. The proportional odds assumption was evaluated using the Brant test and was not violated. *p*-values for CDT models were derived from z-tests. Model fit is reported as R^2^ and adjusted R^2^ for linear models and McFadden’s pseudo-R^2^ for the proportional odds model.

Residual diagnostics (quantile–quantile plots, residuals-versus-fitted plots, and Cook’s distance) were examined for all linear models, and no material violations were identified. Prior to model specification, pairwise associations among candidate covariates were examined using Kendall’s τ rank correlations (Supplement File, Figure S1) to avoid redundant predictors and unstable weights.

Three pre-specified models of increasing adjustment were estimated for each outcome. Model 1 was unadjusted. Model 2 adjusted for age and sex. Model 3 combined inverse probability weighting (IPW) with regression adjustment for age and sex, implementing a doubly robust estimator. Propensity scores were estimated using the covariate- balancing propensity score (CBPS) method [34], targeting the average treatment effect in the treated (ATT). The propensity model included BMI, smoking status, educational attainment, and comorbidity status (any vs. none) variables with adequate distributional overlap between groups that represent plausible antecedent confounders. Hematological parameters were excluded because they are direct physiological consequences of β-thalassemia rather than confounders; their near-complete separation between groups would render weighting degenerate. Serum ferritin was excluded for the same reasons and additionally because it lies on the causal pathway from disease to cognitive outcome; its inclusion would therefore constitute adjustment for a mediator rather than a confounder. Weights were truncated at the 99th percentile. Covariate balance was assessed by standardized mean differences (SMD), with SMD < 0.1 as the adequacy threshold. All four covariates achieved SMD = 0.00 after weighting (Supplement File, Figure S2). The effective control sample size decreased from 30 to 16.4 after weighing; Model 3 estimates should therefore be interpreted as a sensitivity analysis reflecting the direction and approximate magnitude of confounding rather than as primary inferential results. Residual confounding by unmeasured variables, including premorbid intelligence, occupational complexity, and socioeconomic position, cannot be excluded. The IPW-weighted CDT model was estimated using the svyolr function from the survey package [35] to handle fractional weights correctly. The case–control and cross-sectional design preclude causal interpretation regardless of the analytical approach employed.

To characterize the analytical sensitivity of the achieved sample, a post hoc power analysis was conducted for the primary outcome (MoCA total score) using a two-sided independent-samples *t*-test at α = 0.05. With 50 cases and 30 controls and the observed standard deviations (4.49 and 2.82, respectively), the study had 80% power to detect a mean difference of approximately 2.3 points, which corresponds to the minimally important difference commonly cited for MoCA in clinical populations. For multivariable regression models, the effective covariate-to-observation ratio was maintained within acceptable limits for the linear models. The IPW-weighted models were based on a reduced effective control sample size of 16.4, which constrains precision and is explicitly acknowledged in the interpretation of those estimates.

No formal adjustment for multiple comparisons was applied. Given the number of outcomes and models evaluated, the risk of type I error inflation should be considered, and findings are interpreted as hypothesis-generating rather than confirmatory.

All analyses were conducted in R version 4.5.2 [36] using kkstatfun [37], WeightIt [38], cobalt [39], MASS [40], and survey [35]. Statistical significance was defined at α = 0.05.

## 3. Results

### 3.1. Demographic and Clinical Characteristics

A total of 80 individuals were included in the study: 50 patients with transfusion-dependent β-thalassemia and 30 healthy controls. The two groups were comparable with respect to age, sex distribution, marital status, and educational attainment (Table 1). A description of the disease-related clinical features of the patients is provided in Supplement File, Table S1, and the distribution of variables by group is shown in the Supplement File, Figure S3.

Patients exhibited significantly lower height, weight, and body mass index than controls. Smoking was more frequent among controls, whereas the prevalence of comorbid conditions and prior thrombotic events did not differ significantly between groups. Marked hematological differences were present. Patients demonstrated substantially lower hemoglobin concentration, red blood cell count, and hematocrit, together with higher platelet and white blood cell counts. Indices of red cell morphology (MCV and MCH) were also reduced in patients.

Patients more frequently described having overprotective parenting during childhood compared with controls (38.0% vs. 16.7%, *p* = 0.044). Feelings of inferiority attributed to the disease were reported exclusively among patients (16.0% vs. 0.0%, *p* = 0.022). School absenteeism was markedly more common in the β-thalassemia group (*p* < 0.001), reflecting the practical impact of repeated hospital visits and transfusion schedules.

Self-reported memory problems were more frequent among patients, although the difference did not reach statistical significance (*p* = 0.325). Diagnosed depression was reported only in patients, but the small number of events limited statistical power (*p* = 0.151).

### 3.2. Regression Models: Effect of β-Thalassemia on Cognitive Outcomes

**Figure 1:**
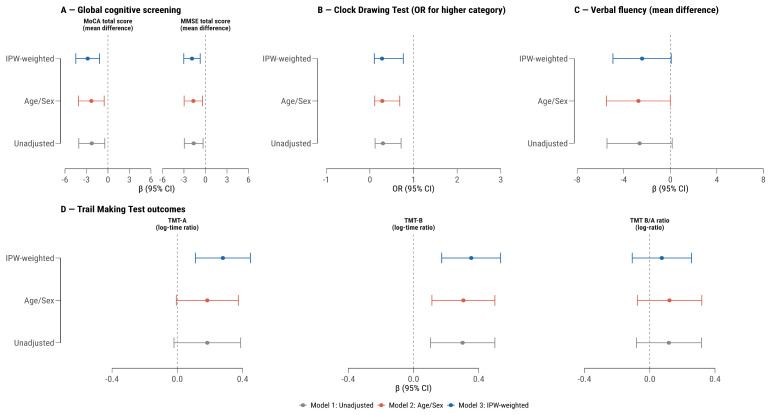
Association between transfusion-dependent β-thalassemia and cognitive performance across three regression models. Forest plots show effect estimates and 95% confidence intervals for the association between β-thalassemia status (reference = controls) and cognitive outcomes. For MoCA, MMSE, and verbal fluency, negative coefficients indicate poorer performance in patients. For the Trail Making Test measures, positive coefficients indicate longer completion times (worse performance). For the Clock Drawing Test, results are presented as odds ratios, with OR < 1 indicating lower odds of belonging to a higher performance category in patients.

A summary of regression coefficients across all outcomes and models is presented in Figure 1. Models estimated for each outcome, alongside model fit statistics, are provided in Table 2.

Across all cognitive outcomes, the direction of effect estimates was consistent from Model 1 (unadjusted) through Model 2 (demographic adjustment for age and sex) to Model 3 (doubly robust IPW-weighted). Adjustment for age and sex in Model 2 produced negligible changes in point estimates relative to Model 1 for all outcomes, indicating that the demographic composition of the two groups did not materially confound the observed associations. In Model 3, which additionally balanced BMI, smoking, educational attainment, and comorbidity through covariate-balancing propensity score weighting, effect estimates were modestly larger than in Models 1 and 2 for most outcomes, most notably for MoCA total score (from −2.26 unadjusted to −2.83 IPW-weighted), TMT-A (time ratio 1.20 to 1.32), and TMT-B (time ratio 1.35 to 1.42). This pattern indicates that the measured confounders included in the propensity model were not masking the association between thalassemia status and cognitive performance; rather, their distribution slightly favoured the patient group on factors associated with worse cognition (e.g., lower BMI, lower smoking prevalence), and balancing these characteristics modestly amplified the estimated differences. The one exception was verbal fluency, for which the IPW-weighted estimate was marginally attenuated relative to the unadjusted model (−2.64 to −2.43 words), though all three estimates remained non-significant and closely clustered. The stability and directional consistency of estimates across models, including the doubly robust specification, supports the robustness of the observed associations to the measured confounders incorporated in the analysis.

#### 3.2.1. MoCA Total Score

Transfusion-dependent β-thalassemia was associated with significantly lower MoCA total scores compared with controls across all three models. In the unadjusted analysis, the mean difference was −2.26 points (95% CI: −4.08 to −0.44; *p* = 0.016; R^2^ = 0.073). After adjustment for age and sex, the estimated difference was −2.32 points (95% CI: −4.12 to −0.52; *p* = 0.012; adjusted R^2^ = 0.084), indicating that demographic factors did not attenuate the group difference. In the IPW doubly robust model, which additionally balanced BMI, smoking status, educational attainment, and comorbidity through propensity weighting, the estimated difference was −2.83 points (95% CI: −4.49 to −1.17; *p* = 0.001; adjusted R^2^ = 0.148). Effect estimates were slightly larger in the doubly robust model compared with the unadjusted analysis.

#### 3.2.2. MMSE Total Score

Patients with transfusion-dependent β-thalassemia had significantly lower MMSE total scores compared with controls across all analytical models. In the unadjusted analysis, the mean difference was −1.64 points (95% CI: −2.95 to −0.33; *p* = 0.015; R^2^ = 0.074). Adjustment for age and sex yielded a similar estimate of −1.69 points (95% CI: −2.98 to −0.40; *p* = 0.011; adjusted R^2^ = 0.096). In the IPW-weighted doubly robust model, the estimated difference was −1.87 points (95% CI: −3.02 to −0.73; *p* = 0.002; adjusted R^2^ = 0.153). Effect estimates were consistent across all three models, with no attenuation following adjustment. Although statistically significant, the magnitude of the MMSE deficit was smaller than that observed for the MoCA. Using the conventional MoCA threshold of less than 26 points, 56.0% of patients with transfusion-dependent β-thalassemia were classified as impaired, compared with 30.0% of controls (OR = 2.93; 95% CI: 1.04–8.82; *p* = 0.037). Using the conventional MMSE threshold of less than 24 points, 10.0% of patients scored below the impairment cut-off, compared with 0.0% of controls.

#### 3.2.3. Clock Drawing Test

Patients with transfusion-dependent β-thalassemia showed significantly lower CDT performance across all three models. In the unadjusted analysis, the odds ratio was 0.30 (95% CI: 0.12 to 0.72; *p* = 0.008; McFadden pseudo-R^2^ = 0.034), indicating that patients had approximately 70% lower odds of achieving higher visuospatial–executive performance categories compared with controls. Adjustment for age and sex yielded a comparable estimate (OR 0.28; 95% CI: 0.11 to 0.69; *p* = 0.007; pseudo-R^2^ = 0.043). In the IPW-weighted model, estimated using survey-weighted ordinal regression to accommodate fractional propensity weights, the association remained statistically significant (OR 0.28; 95% CI: 0.10 to 0.77; *p* = 0.013). The magnitude of the association was consistent across models, and no meaningful attenuation was observed following demographic or propensity adjustment.

#### 3.2.4. Trail Making Test Outcomes

For TMT-A, the unadjusted log-ratio was 0.184 (95% CI: −0.020 to 0.388; *p* = 0.076), corresponding to an estimated 20% longer completion time among patients (exp(β) = 1.20; 95% CI: 0.98 to 1.47), which did not reach conventional statistical significance. After adjustment for age and sex, the estimate was unchanged (log-ratio 0.184; exp(β) = 1.20; 95% CI: 0.99 to 1.45; *p* = 0.058). In the IPW-weighted model, the association reached statistical significance (log-ratio 0.279; exp(β) = 1.32; 95% CI: 1.12 to 1.56; *p* = 0.001; adjusted R^2^ = 0.268), corresponding to a 32% longer completion time among patients in the weighted model.

For TMT-B, the between-group difference was larger and statistically significant across all models. The unadjusted log-ratio was 0.301 (exp(β) = 1.35; 95% CI: 1.11 to 1.65; *p* = 0.003; R^2^ = 0.106), indicating that patients required approximately 35% longer to complete the task. Demographic adjustment yielded a nearly identical estimate (log-ratio 0.306; exp(β) = 1.36; 95% CI: 1.12 to 1.65; *p* = 0.002; adjusted R^2^ = 0.140). In the IPW-weighted model, the association was slightly amplified (log-ratio 0.354; exp(β) = 1.42; 95% CI: 1.19 to 1.71; *p* < 0.001; adjusted R^2^ = 0.247), indicating a 42% longer completion time among patients after balancing measured covariates.

The TMT B/A ratio did not demonstrate statistically significant group differences in any model. The unadjusted log-ratio was 0.117 (exp(β) = 1.12; 95% CI: 0.92 to 1.37; *p* = 0.245), with similar estimates after demographic adjustment (0.121; exp(β) = 1.13; 95% CI: 0.93 to 1.38; *p* = 0.224) and further attenuation under IPW (0.074; exp(β) = 1.08; 95% CI: 0.90 to 1.29; *p* = 0.418). Model R^2^ values for the ratio were uniformly low (0.017–0.069).

#### 3.2.5. Verbal Fluency

Verbal fluency performance was lower in patients with transfusion-dependent β-thalassemia across all three models; however, the differences did not reach conventional statistical significance. In the unadjusted analysis, patients produced on average 2.64 fewer words than controls (95% CI: −5.44 to 0.16; *p* = 0.064; R^2^ = 0.043). After adjustment for age and sex, the estimated difference was −2.74 words (95% CI: −5.49 to 0.00; *p* = 0.050; adjusted R^2^ = 0.069), reaching the boundary of statistical significance. In the IPW-weighted model, the difference was −2.43 words (95% CI: −4.92 to 0.07; *p* = 0.057; adjusted R^2^ = 0.076). Verbal fluency estimates were relatively stable across models, suggesting that the balanced covariates do not substantially confound this association; the borderline significance across all three models warrants cautious interpretation given the modest sample size.

## 4. Discussion

The present findings demonstrate consistent differences in cognitive screening performance between adults with transfusion-dependent β-thalassemia and frequency-matched healthy controls. These differences were observed across multiple domains: global cognition, visuospatial, executive function, and processing speed. Importantly, these associations remained stable or amplified across unadjusted, demographically adjusted, and propensity-weighted analytical models. The consistency of findings across analytical approaches supports a robust association between transfusion-dependent β-thalassemia and multidomain cognitive vulnerability in adulthood.

### 4.1. Global Cognitive Performance

The present study demonstrated consistently lower global cognitive screening performance in adults with transfusion-dependent β-thalassemia compared with frequency-matched healthy controls. This pattern was evident for both the MoCA and MMSE and remained stable after demographic adjustment and propensity-weighted analysis, indicating that the observed differences were not explained by measured confounders included in the models.

The MoCA appeared more sensitive than the MMSE in detecting between-group differences, which is consistent with the prior literature showing superior sensitivity of the MoCA for milder deficits, particularly in executive and visuospatial domains [22,23,41]. In the present cohort, more than half of patients met the conventional MoCA threshold for impairment, whereas a substantially smaller proportion fell below the MMSE threshold. This discordance supports the interpretation that the MMSE may underestimate subtle or domain-specific deficits in younger adult thalassemia populations.

These findings are broadly consistent with previous studies reporting lower cognitive performance in adult thalassemia cohorts [15,16,18,42]. At the same time, prevalence estimates across studies vary considerably, likely because of differences in age structure, disease severity, cognitive instruments, and case definitions. Importantly, MoCA and MMSE are screening tools rather than diagnostic neuropsychological instruments, and the present findings should therefore be interpreted as evidence of lower cognitive screening performance rather than definitive clinical neurocognitive disorder. Nevertheless, the magnitude and consistency of the observed MoCA difference suggest that global cognitive vulnerability in transfusion-dependent adults is clinically relevant and merits attention in routine follow-up.

### 4.2. Domain-Specific Findings

The domain-specific results suggest that the cognitive profile associated with transfusion-dependent β-thalassemia is driven more strongly by executive and visuospatial difficulties than by isolated slowing alone. CDT performance was consistently poorer in patients across all analytical models, with little attenuation after adjustment. This stability supports the robustness of the observed association and is in line with earlier reports identifying visuospatial and executive dysfunction as salient features of cognitive vulnerability in thalassemia [10,14,15].

A similar pattern emerged in the Trail Making Test, though its interpretation warrants caution. TMT-B, which places greater demands on set-shifting and executive control, showed larger and statistically significant between-group differences across all three models. TMT-A, which primarily reflects visuomotor scanning and basic processing speed, showed a smaller and nominally non-significant difference in the unadjusted and demographically adjusted models, but reached statistical significance in the IPW-weighted model. The greater magnitude of impairment on TMT-B could be interpreted as evidence of selective executive vulnerability. However, cognitive fatigue represents an equally compelling alternative explanation. Patients with transfusion-dependent β-thalassemia are exposed to multiple conditions known to produce neuroasthenia and performance decrements on high-effort tasks, including chronic anaemia, systemic iron overload, sleep disturbance, and depression. Under a fatigue model, basic low-effort tasks such as TMT-A would be expected to show smaller and less consistent effects than the cognitively demanding set-shifting required by TMT-B, precisely the pattern observed in Models 1 and 2. The present data cannot distinguish between these two mechanisms in the absence of objective fatigue measures, and reliance on a single testing occasion preclude causal attribution. The finding that TMT-A also reached significance after propensity weighting in Model 3, and that CDT performance was consistently impaired across all models under relatively low-demand visuospatial conditions, offers partial evidence against a pure fatigue account, but does not exclude it. Both executive dysfunction and cognitive fatigue secondary to chronic disease burden should be regarded as plausible and non-mutually exclusive contributors to the observed TMT profile, and future studies incorporating objective fatigue assessments and longitudinal cognitive monitoring are needed to resolve this question. The absence of a significant difference in the TMT B/A ratio is consistent with prior work, suggesting that this derived measure may add limited interpretive value beyond the individual subtests [43].

Verbal fluency performance was modestly lower in patients; however, there was no clear evidence of a statistically robust difference between groups. Estimates were consistently in the same direction across models but remained borderline in significance. Given the modest sample size and the known sensitivity of verbal fluency to educational and sociocultural factors [44], these findings should be interpreted cautiously and do not support a definitive difference in this domain.

### 4.3. Pathophysiological Considerations

Several biological mechanisms may plausibly contribute to lower cognitive test performance in transfusion-dependent β-thalassemia; however, these interpretations remain hypothesis-generating, as the present study did not include direct measures of brain structure, cerebral iron deposition, or hemoglobin levels at the time of cognitive testing.

Systemic iron overload has been associated with lower cognitive performance, potentially through oxidative stress, mitochondrial dysfunction, lipid peroxidation, and neuroinflammation [45,46]. Transfusion-dependent patients accumulate systemic iron through both transfusion-related loading and increased intestinal absorption. Prior studies have reported associations between serum ferritin, chelation non-compliance, and poorer cognitive outcomes [18,47], although ferritin is a systemic marker that does not directly reflect cerebral iron deposition, and neuroimaging data would be required to confirm brain-specific iron burden. The hypercoagulable state in β-thalassemia may increase the risk of silent cerebrovascular injury [19], although this remains speculative without neuroimaging. Psychosocial and disease-burden factors may additionally contribute to the observed performance differences through mechanisms other than structural neurological injury. Chronic anaemia impairs cerebral oxygen delivery; systemic iron overload is associated with oxidative stress and neuroinflammation; and conditions including sleep disturbance, depression, and chronic pain are prevalent in transfusion-dependent populations and are each capable of producing neuroasthenia and reversible cognitive performance decrements, particularly on high-effort tasks [48,49]. In the present cohort, 10.0% of patients had diagnosed depression compared with none of the controls, but concurrent standardised assessment of fatigue, sleep quality, or mood was not performed. The absence of these measures represents a meaningful limitation, as it precludes quantification of the contribution of reversible fatigue-related mechanisms to the observed cognitive profile and limits the extent to which the findings can be attributed to fixed organic dysfunction rather than state-dependent performance variability.

In addition, patients more frequently reported overprotective parenting and illness-related feelings of inferiority; although not included in the analytical models, these variables may reflect early-life influences on cognitive development and cognitive reserve.

Taken together, these mechanisms should be interpreted as conceptual frameworks for future investigation rather than as explanations supported by direct evidence from the present data.

### 4.4. Clinical Implications

The high prevalence of MoCA-defined lower cognitive performance in an adult transfusion-dependent cohort may have important implications for clinical practice. Cognitive dysfunction has the capacity to influence treatment adherence, chelation compliance, vocational functioning, and overall quality of life. Incorporating brief cognitive screening into routine adult thalassemia follow-up may therefore be warranted. The data support the use of the MoCA as a preferred screening instrument in this setting, given its greater sensitivity relative to the MMSE. Supplementary executive measures such as the Clock Drawing Test or Trail Making Test may provide additional clinically relevant information. Early identification of cognitive vulnerability could facilitate targeted neuropsychological evaluation, patient education, and optimization of modifiable risk factors, including iron management and vascular risk control.

### 4.5. Limitations

Several limitations should be considered when interpreting these findings.

First, the case–control and cross-sectional design preclude causal inference. Cognitive function and disease status were measured at a single time point, and the temporal sequence between β-thalassemia and cognitive differences cannot be established. Prospective longitudinal studies with repeated cognitive assessment are required to characterize the natural history of cognitive test performance in this population.

Second, the use of hospital-based controls may introduce selection bias, as such individuals may differ from the general population in health-seeking behavior or unmeasured socioeconomic factors. Although major medical and neurological conditions were actively excluded, residual differences cannot be ruled out. However, recruitment from the same clinical setting ensured standardized assessment conditions and reduced the risk of differential measurement bias between groups.

Third, haemoglobin concentration at the time of cognitive testing was not directly measured, as only pre-transfusion values were available, and no concurrent assessment of oxygenation status, whether by same-session haemoglobin sampling or pulse oximetry, was performed during cognitive assessment. This represents a meaningful methodological limitation. Haemoglobin is a primary determinant of cerebral oxygen delivery, and inter-individual variability in post-transfusion haemoglobin response, which is well-documented in transfusion-dependent populations, may have introduced unmeasured physiological heterogeneity at the time of testing. Cognitive performance, particularly on high-effort tasks such as TMT-B, is sensitive to acute fluctuations in cerebral oxygenation, and the absence of a concurrent oxygenation measure precludes adjustment for this source of variability. The temporal separation between pre-transfusion haematological measurements and post-transfusion cognitive assessment therefore limits the extent to which the observed between-group differences can be attributed to stable trait-level cognitive differences rather than to state-dependent physiological variation. The 48–72 h post-transfusion window was selected to ensure haemodynamic stability and minimise the acute effects of the transfusion procedure itself; however, future studies in this population should incorporate same-session haemoglobin measurement or continuous oxygenation monitoring to enable direct evaluation of the haemoglobin–cognition relationship within patients.

Fourth, the overall sample size was modest. The reduction in the effective control sample from 30 to 16.4 under propensity weighting substantially reduces precision; IPW results should therefore be interpreted as sensitivity analyses rather than confirmatory estimates. The sample was additionally insufficient to examine cognitive outcomes by thalassemia subtype: 86% of patients had thalassemia major, and 14% had transfusion-dependent intermedia, precluding assessment of whether cognitive profiles differ between these phenotypes. In addition, no formal correction for multiple testing was applied, and the potential for type I error inflation should be considered when interpreting the results.

Fifth, residual confounding cannot be excluded. Serum ferritin was excluded from all models because it is a downstream disease consequence and a potential mediator rather than a confounder, and because near-complete separation between groups violates the positivity assumption; the ferritin–cognition relationship therefore requires separate within-patient investigation. Endocrine and metabolic complications of thalassemia were captured only as a binary composite comorbidity variable, which is insufficiently granular to control for independent cognitive effects of conditions such as hypothyroidism, hypogonadism, diabetes mellitus, and chronic liver disease.

Sixth, cognitive assessments were not conducted by evaluators blinded to group assignments, introducing potential information bias, particularly for timed tasks. Although standardized protocols were followed, assessor awareness may have influenced administration. Normative Bulgarian population data are additionally limited for several instruments, making absolute performance benchmarking difficult.

Seventh, the instruments used were screening tools rather than comprehensive neuropsychological assessments and are not diagnostic of clinical neurocognitive disorder. MoCA, MMSE, CDT, TMT, and verbal fluency identify possible lower performance but cannot establish the presence, etiology, or clinical significance of cognitive impairment.

Eighth, single-center recruitment limits generalizability, and the predominance of thalassemia major restricts inference to more severe phenotypes.

Despite these limitations, this study addresses a current gap in the literature by focusing on adults with transfusion-dependent β-thalassemia and adopting a multidomain cognitive battery. The analytical strategy incorporated both conventional regression adjustment and covariate balancing propensity score weighting, yielding a doubly robust framework that enhances internal validity under measured confounding.

## 5. Conclusions

Adults with transfusion-dependent β-thalassemia showed lower cognitive performance across multiple domains, particularly on executive and visuospatial tasks. These differences remained consistent across unadjusted, demographically adjusted, and propensity-weighted analyses, were not explained by measured confounders, and demonstrate associations robust to analytical approach. However, these findings represent associative evidence from cross-sectional screening data and do not establish irreversible cognitive impairment, causality, or clinical neurocognitive disorder. Longitudinal studies incorporating serial cognitive testing, neuroimaging biomarkers, and quantitative measures of iron burden are needed to clarify the mechanisms, trajectory, reversibility, and clinical significance of the observed differences.

## Figures and Tables

**Table 1:** Baseline demographic, clinical, laboratory, and hemostatic characteristics of study participants.

Characteristic *	β-Thalassemia (n = 50)	Controls (n = 30)	*p*-Value
Demographics and lifestyle			
Age (years)	35.74 (12.74)	35.67 (13.42)	>0.90
Female sex	20 (40.0%)	11 (36.7%)	0.767
Married	17 (34.0%)	10 (33.3%)	>0.90
Below university education	35 (70.0%)	17 (56.7%)	0.226
Height (cm)	167.52 (9.38)	176.07 (11.55)	0.001
Weight (kg)	62.58 (11.85)	79.60 (21.05)	<0.001
Body mass index (kg/m^2^)	22.23 (3.29)	25.56 (6.13)	0.009
Current smoking	15 (30.0%)	17 (56.7%)	0.018
Comorbidity (any)	22 (44.0%)	10 (33.3%)	0.346
Prior thrombotic event	1 (2.0%)	0 (0.0%)	>0.90
Hematological and biochemical parameters			
Hemoglobin (g/L)	82.58 (11.29)	151.07 (12.24)	<0.001
RBC count (×10^12^/L)	3.31 (0.77)	5.06 (0.42)	<0.001
Hematocrit	0.27 (0.04)	0.45 (0.04)	<0.001
WBC count (×10^9^/L)	12.99 (6.97)	7.06 (1.57)	<0.001
Platelets (×10^9^/L)	577.30 (305.70)	231.70 (36.48)	<0.001
MCV (fL)	83.45 (6.86)	88.59 (7.23)	0.003
MCH (pg)	26.44 (2.38)	29.80 (1.99)	<0.001
MCHC (g/L)	303.41 (47.62)	324.66 (59.10)	0.101
AST (U/L)	56.34 (104.05)	23.05 (10.72)	0.029
ALT (U/L)	34.13 (33.82)	39.33 (24.34)	0.428
Ferritin (ng/mL)	1217.11 (1642.13)	114.56 (85.13)	<0.001
HBsAg positive	1 (2.0%)	1 (3.3%)	>0.90
Anti-HCV positive	3 (6.0%)	0 (0.0%)	0.288
Self-reported Psychosocial Characteristics			
Overprotective parents	19 (38.0%)	5 (16.7%)	0.044
Self-reported memory problems	15 (30.0%)	6 (20.0%)	0.325
Feelings of inferiority due to illness	8 (16.0%)	0 (0.0%)	0.022
Diagnosed depression	5 (10.0%)	0 (0.0%)	0.151
Frequent school absence	17 (34.0%)	0 (0.0%)	<0.001

* Values are presented as mean (standard deviation) for continuous variables and number (percentage) for categorical variables. *p*-values were calculated using independent-samples *t*-tests for continuous variables and χ^2^ or Fisher’s exact tests for categorical variables. Abbreviations: RBC, red blood cell; WBC, white blood cell; MCV, mean corpuscular volume; MCH, mean corpuscular hemoglobin; MCHC, mean corpuscular hemoglobin concentration; AST, aspartate aminotransferase; ALT, alanine aminotransferase; HBsAg, hepatitis B surface antigen; HCV, hepatitis C virus.

**Table 2:** Effect of β-thalassemia on cognitive outcomes. Regression coefficients with 95% confidence intervals across three analytical models.

Outcome	Model 1 Unadjusted	Model 2 Demographic Adjustment	Model 3 IPW-Weighted
Model estimates			
MoCA total score	β = −2.26 (−4.08, −0.44);	β = −2.32 (−4.12, −0.52);	β = −2.83 (−4.49, −1.17);
	*p* = 0.016	*p* = 0.012	*p* = 0.001
MMSE total score	β = −1.64 (−2.95, −0.33);	β = −1.69 (−2.98, −0.40);	β = −1.87 (−3.02, −0.73);
	*p* = 0.015	*p* = 0.011	*p* = 0.002
CDT ^a^	OR = 0.30 (0.12, 0.72);	OR = 0.28 (0.11, 0.69);	OR = 0.28 (0.10, 0.77);
	*p* = 0.008	*p* = 0.007	*p* = 0.013
TMT-A ^b^	β = 0.184 (−0.020, 0.388);	β = 0.184 (−0.006, 0.374);	β = 0.279 (0.111, 0.448);
	*p* = 0.076;	*p* = 0.058;	*p* = 0.001;
	Ratio = 1.20 (0.98, 1.47)	Ratio = 1.20 (0.99, 1.45)	Ratio = 1.32 (1.12, 1.56)
TMT-B ^b^	β = 0.301 (0.104, 0.499);	β = 0.306 (0.113, 0.499);	β = 0.354 (0.173, 0.534);
	*p* = 0.003;	*p* = 0.002;	*p* < 0.001;
	Ratio = 1.35 (1.11, 1.65)	Ratio = 1.36 (1.12, 1.65)	Ratio = 1.42 (1.19, 1.71)
TMT B/A ratio ^b^	β = 0.117 (−0.082, 0.317);	β = 0.121 (−0.076, 0.319);	β = 0.074 (−0.107, 0.256);
	*p* = 0.245;	*p* = 0.224;	*p* = 0.418;
	Ratio = 1.12 (0.92, 1.37)	Ratio = 1.13 (0.93, 1.38)	Ratio = 1.08 (0.90, 1.29)
Verbal fluency (words)	β = −2.64 (−5.44, 0.16);	β = −2.74 (−5.49, 0.00);	β = −2.43 (−4.92, 0.07);
	*p* = 0.064	*p* = 0.050	*p* = 0.057
Model diagnostics			
MoCA	R^2^ = 0.073; adj. R^2^ = 0.061	R^2^ = 0.119; adj. R^2^ = 0.084	R^2^ = 0.180; adj. R^2^ = 0.148
MMSE	R^2^ = 0.074; adj. R^2^ = 0.062	R^2^ = 0.130; adj. R^2^ = 0.096	R^2^ = 0.186; adj. R^2^ = 0.153
CDT	pseudo-R^2^ = 0.034	pseudo-R^2^ = 0.043	—
TMT-A	R^2^ = 0.040; adj. R^2^ = 0.027	R^2^ = 0.187; adj. R^2^ = 0.155	R^2^ = 0.296; adj. R^2^ = 0.268
TMT-B	R^2^ = 0.106; adj. R^2^ = 0.094	R^2^ = 0.172; adj. R^2^ = 0.140	R^2^ = 0.276; adj. R^2^ = 0.247
TMT B/A ratio	R^2^ = 0.017; adj. R^2^ = 0.005	R^2^ = 0.064; adj. R^2^ = 0.027	R^2^ = 0.069; adj. R^2^ = 0.033
Verbal fluency	R^2^ = 0.043; adj. R^2^ = 0.031	R^2^ = 0.105; adj. R^2^ = 0.069	R^2^ = 0.112; adj. R^2^ = 0.076

^a^ Clock Drawing Test modeled using proportional odds logistic regression (MASS::polr for Models 1–2; survey-weighted svyolr for Model 3). The proportional odds assumption was evaluated using the Brant test and was not violated (omnibus χ^2^ = 7.62, df = 12, *p* = 0.81). Coefficients are reported as odds ratios for belonging to a higher CDT performance category; OR < 1 indicates lower odds of higher performance (worse visuospatial–executive function) in patients relative to controls. *p*-values derived from z-tests. ^b^ TMT-A, TMT-B, and TMT B/A ratio modeled on the log scale. β = log-time ratio; Ratio = exp(β), the multiplicative difference in mean completion time (cases relative to controls). MoCA, MMSE, and verbal fluency modeled on the raw scale; β = mean difference in score. Model 2 adjusts for age and sex. Model 3 combines CBPS-estimated inverse probability weights (ATT estimand, balancing BMI, smoking status, educational attainment, and comorbidity) with regression adjustment for age and sex, yielding a doubly robust estimator. Effective control sample size after weighting: 16.4 (unadjusted: 30).

## Data Availability

The data presented in this study are available on request from the corresponding author due to ethical and legal restrictions related to patient confidentiality and the sensitive nature of rare disease medical data.

## Supplementary Materials

The following supporting information can be downloaded at https://www.mdpi.com/article/10.3390/neurolint18060101/s1, Figure S1. Pairwise Kendall’s τ rank correlation matrix of all study variables (N = 80). Figure S2. Propensity score distributions before and after covariate balancing propensity score (CBPS) inverse probability weighting. Figure S3. Distribution of key clinical and demographic variables by study group (β-thalassemia, n = 50; controls, n = 30). Table S1. Disease-specific clinical, hematological, coagulation, and serological characteristics of patients with transfusion-dependent β-thalassemia (n = 50), stratified by thalassemia subtype (thalassemia major, n = 43; transfusion-dependent intermedia, n = 7).

## Abbreviations

The following abbreviations are used in this manuscript:

ALT	Alanine aminotransferase
aPTT	Activated partial thromboplastin time
AST	Aspartate aminotransferase
ATT	Average treatment effect in the treated
BMI	Body mass index
CBPS	Covariate balancing propensity score
CDT	Clock Drawing Test
CI	Confidence interval
HBB	Hemoglobin subunit beta gene
HBsAg	Hepatitis B surface antigen
HCV	Hepatitis C virus
IPW	Inverse probability weighting
MCH	Mean corpuscular hemoglobin
MCHC	Mean corpuscular hemoglobin concentration
MCV	Mean corpuscular volume
MMSE	Mini-Mental State Examination
MoCA	Montreal Cognitive Assessment
OR	Odds ratio
PLT	Platelet count
RBC	Red blood cell
SD	Standard deviation
SMD	Standardized mean difference
TDT	Transfusion-dependent thalassemia
TMT	Trail Making Test
TMT-A	Trail Making Test, Part A
TMT-B	Trail Making Test, Part B
WBC	White blood cell
