# Supplementary material for: Cognitive Performance in Transfusion-Dependent Adults with β-Thalassemia in Bulgaria: A Case–Control Study
Source: Neurol Int. 2026 May 22;18(6):101. doi: 10.3390/neurolint18060101 (PMC13306042; doi:10.3390/neurolint18060101)

Supplementary File

Cognitive Performance in Transfusion-Dependent Adults with β-Thalassemia in Bulgaria: A Case–Control Study

Viktoria Babacheva, Kostadin Kostadinov, Veselina Goranova-Marinova, Miroslava Hristova, Penka Atanassova

Table of Contents

Figure S1. Pairwise Kendall's τ rank correlation matrix of all study variables (N = 80).

Figure S2. Propensity score distributions before and after covariate balancing propensity score (CBPS) inverse probability weighting.

Figure S3. Distribution of key clinical and demographic variables by study group (β-thalassemia, n = 50; controls, n = 30).

Table S1. Disease-specific clinical, hematological, coagulation, and serological characteristics of patients with transfusion-dependent β-thalassemia (n = 50), stratified by thalassemia subtype (thalassemia major, n = 43; transfusion-dependent intermedia, n = 7).

Figure S1. Pairwise Kendall's τ rank correlation matrix of all study variables. The upper triangle of the matrix displays Kendall's τ coefficients for each variable pair, estimated using pairwise complete observations in the full analytic sample (N = 80). Color intensity reflects the magnitude and direction of the association (blue = negative, red = positive, white = near-zero).

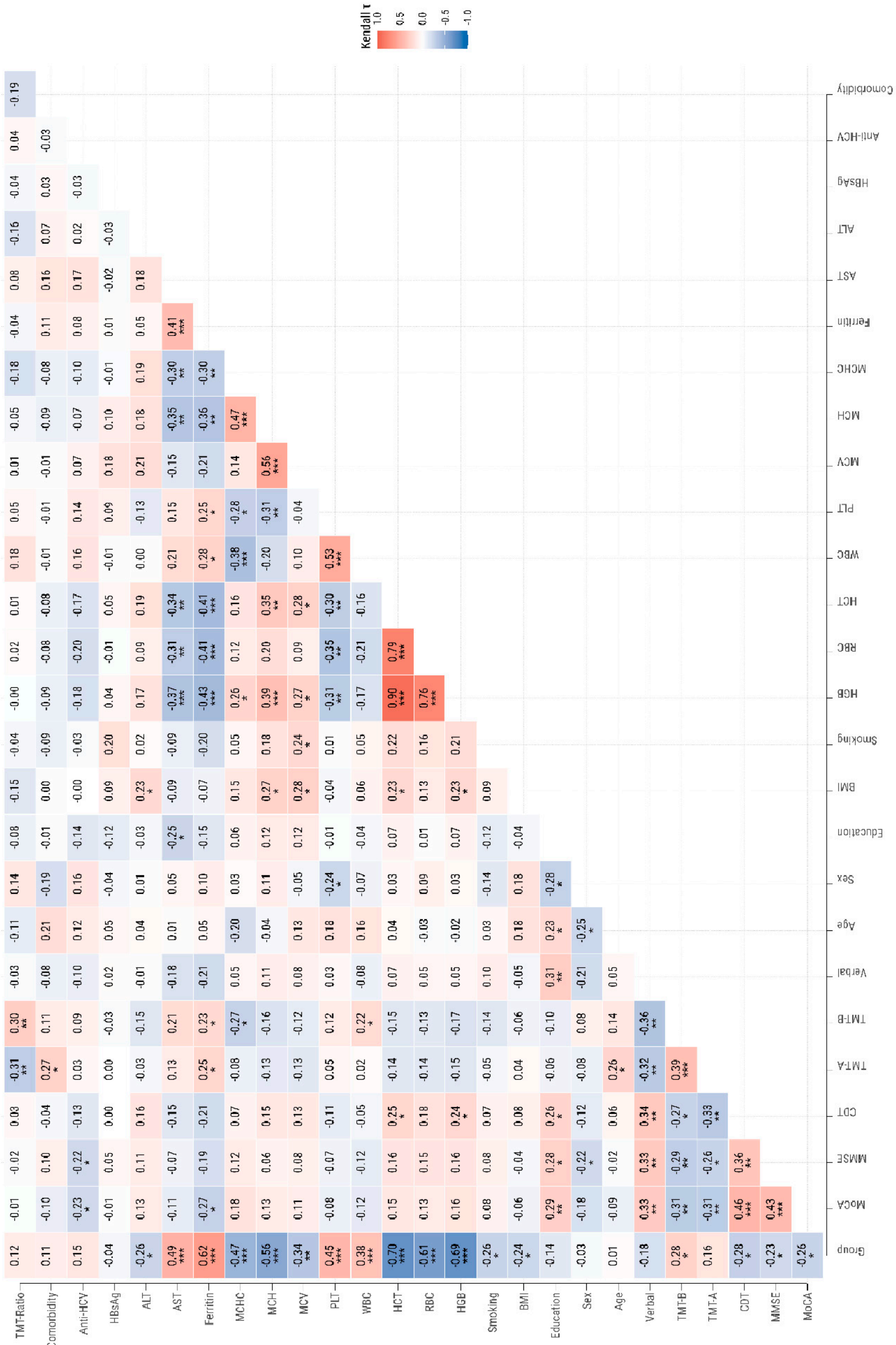

*Asterisks denote statistical significance: *p < 0.05, **p < 0.01, ***p < 0.001; no correction for multiple comparisons was applied. The matrix was examined prior to propensity model specification to characterize the covariate structure and identify potential redundancy among candidate variables. Variables are ordered as*

follows: Group (study group; binary), Age (years), Sex (binary), Education (educational attainment; binary), BMI (body mass index, kg/m^2), Smoking (current smoking status; binary), Comorbidity (any comorbid condition; binary), HGB (hemoglobin, g/L), RBC (red blood cell count, $\times 10^{12}$/L), HCT (hematocrit, fraction), WBC (white blood cell count, $\times 10^9$/L), PLT (platelet count, $\times 10^9$/L), MCV (mean corpuscular volume, fL), MCH (mean corpuscular hemoglobin, pg), MCHC (mean corpuscular hemoglobin concentration, g/L), Ferritin (serum ferritin, ng/mL), AST (aspartate aminotransferase, U/L), ALT (alanine aminotransferase, U/L), HBsAg (hepatitis B surface antigen; binary), Anti-HCV (hepatitis C antibody; binary), MoCA (Montreal Cognitive Assessment total score, 0–30), MMSE (Mini-Mental State Examination total score, 0–30), CDT (Clock Drawing Test, Shulman scoring, 0–5), TMT-A (Trail Making Test Part A completion time, seconds), TMT-B (Trail Making Test Part B completion time, seconds), TMT-Ratio (TMT B/A ratio, dimensionless), Verbal (verbal fluency, word count in 60 seconds). Binary and categorical variables were converted to integer codes prior to analysis.

Figure S2. Propensity score distributions before and after covariate balancing propensity score (CBPS) inverse probability weighting. Kernel density plots show the estimated propensity scores (conditional probability of belonging to the β-thalassemia group given BMI, smoking status, educational attainment, and comorbidity status) separately for patients (orange) and controls (blue). The left panel displays the unweighted distributions; the right panel displays the weighted distributions after applying ATT weights truncated at the 99th percentile. Improved overlap after weighting indicates that the control group was successfully reweighted to resemble the thalassemia group on the included covariates. Effective control sample size after weighting: 16.4 (unadjusted: 30).

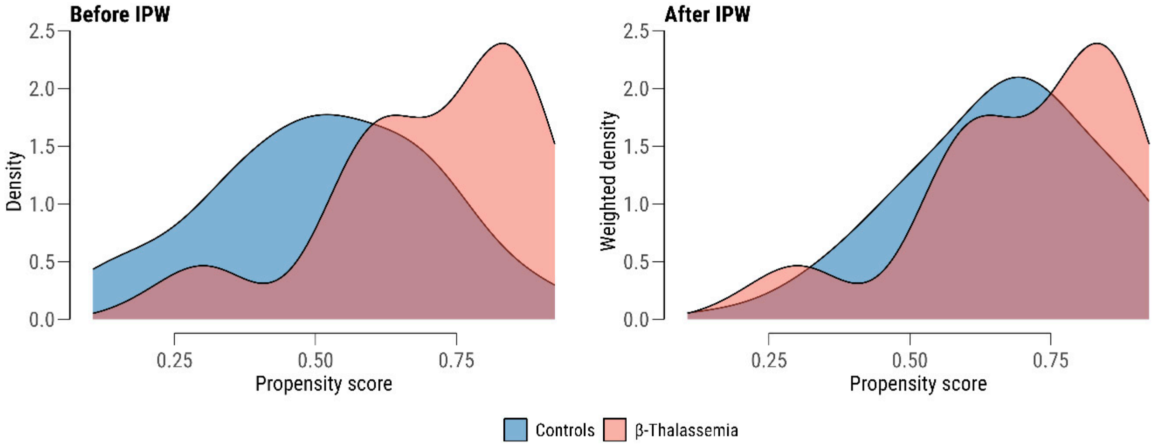

Table S1. Disease-specific clinical, hematological, coagulation, and serological characteristics of patients with transfusion-dependent β-thalassemia (n = 50), stratified by thalassemia subtype.

**Variable**	**All patients (n = 50)**[1]	**Thalassemia major (n = 43)**[1]	**TDT intermedia (n = 7)**[1]	**p-value**[2]
**On chelation therapy**	47 (94.0%)	43 (100.0%)	4 (57.1%)	0.002
**Chelator type**				0.026
Deferasirox	25 (53.2%)	24 (55.8%)	1 (25.0%)	
Deferiprone	17 (36.2%)	16 (37.2%)	1 (25.0%)	
Deferoxamine	3 (6.4%)	1 (2.3%)	2 (50.0%)	
Deferoxamine + Deferiprone	2 (4.3%)	2 (4.7%)	0 (0.0%)	
**Annual transfusion volume (mL/year)**				<0.001
Mean (SD)	6,125.68 (2,585.70)	6,668.81 (2,292.65)	2,789.29 (1,645.16)	
Median (Q1, Q3)	6,319.00 (4,260.00, 8,520.00)	6,319.00 (4,260.00, 8,520.00)	2,130.00 (1,420.00, 4,260.00)	
Min, Max	1,065.00, 14,200.00	3,195.00, 14,200.00	1,065.00, 4,970.00	

**Annual transfusion requirement (units/year)**				<0.001
Mean (SD)	17.24 (7.31)	18.77 (6.49)	7.86 (4.63)	
Median (Q1, Q3)	18.00 (12.00, 24.00)	18.00 (12.00, 24.00)	6.00 (4.00, 12.00)	
Min, Max	3.00, 40.00	9.00, 40.00	3.00, 14.00	
**Splenectomy**	35 (70.0%)	30 (69.8%)	5 (71.4%)	>0.999
**Age at diagnosis (years)**				0.142
Mean (SD)	33.02 (11.70)	31.51 (10.17)	42.29 (16.67)	
Median (Q1, Q3)	31.00 (25.00, 40.00)	30.00 (24.00, 37.00)	48.00 (30.00, 53.00)	
Min, Max	11.00, 65.00	18.00, 65.00	11.00, 60.00	
Min, Max	21.90, 34.90	21.90, 34.90	24.70, 30.90	
**Hemoglobin (g/L)**				0.561
Mean (SD)	82.58 (11.29)	82.16 (11.24)	85.14 (12.17)	
Median (Q1, Q3)	84.00 (75.00, 90.00)	84.00 (75.00, 89.00)	90.00 (74.00, 96.00)	
Min, Max	46.00, 107.00	46.00, 107.00	64.00, 97.00	
**RBC count (×10^{12}/L)**				0.015
Mean (SD)	3.31 (0.77)	3.17 (0.67)	4.19 (0.82)	
Median (Q1, Q3)	3.18 (2.81, 3.50)	3.08 (2.73, 3.46)	3.95 (3.44, 4.87)	
Min, Max	2.25, 5.66	2.25, 5.66	3.34, 5.48	
**Hematocrit (fraction)**				0.082
Mean (SD)	0.27 (0.04)	0.26 (0.04)	0.30 (0.04)	
Median (Q1, Q3)	0.27 (0.24, 0.30)	0.26 (0.23, 0.29)	0.31 (0.25, 0.32)	
Min, Max	0.18, 0.37	0.18, 0.37	0.23, 0.35	
**WBC count (×10^9/L)**				0.719
Mean (SD)	12.99 (6.97)	12.81 (6.72)	14.12 (8.91)	
Median (Q1, Q3)	13.31 (7.03, 16.28)	13.60 (7.03, 16.28)	11.97 (6.70, 20.82)	
Min, Max	2.66, 33.04	2.66, 33.04	5.25, 30.76	
**Platelet count (×10^9/L)**				0.990
Mean (SD)	577.30 (305.70)	577.53 (308.36)	575.86 (312.37)	
Median (Q1, Q3)	597.50 (291.00, 842.00)	590.00 (291.00, 854.00)	614.00 (189.00, 763.00)	
Min, Max	32.00, 1,244.00	32.00, 1,244.00	161.00, 1,033.00	
**MCV (fL)**				0.022
Mean (SD)	83.45 (6.86)	84.81 (5.57)	74.17 (8.08)	
Median (Q1, Q3)	85.60 (80.80, 88.00)	86.80 (81.90, 88.00)	73.65 (67.70, 77.50)	
Min, Max	64.10, 97.40	64.10, 97.40	64.80, 87.70	
**MCH (pg)**				0.022
Mean (SD)	26.44 (2.38)	26.84 (2.02)	22.43 (2.18)	
Median (Q1, Q3)	26.70 (25.05, 28.10)	26.95 (25.70, 28.35)	22.40 (20.95, 23.90)	
Min, Max	19.80, 30.60	21.00, 30.60	19.80, 25.10	
**MCHC (g/L)**				0.027
Mean (SD)	303.41 (47.62)	306.20 (50.72)	286.29 (10.59)	
Median (Q1, Q3)	308.50 (295.00, 322.00)	312.00 (302.00, 326.00)	287.00 (276.00, 295.00)	
Min, Max	3.60, 358.00	3.60, 358.00	273.00, 301.00	
**Serum ferritin (ng/mL)**				0.164
Mean (SD)	1,217.11 (1,642.13)	1,103.61 (1,681.14)	1,914.30 (1,256.64)	
Median (Q1, Q3)	624.70 (399.60, 1,540.00)	557.30 (393.50, 1,316.30)	2,183.00 (587.10, 2,934.00)	
Min, Max	28.00, 10,500.00	108.60, 10,500.00	28.00, 3,429.00	
**AST (U/L)**				0.991
Mean (SD)	56.34 (104.05)	56.37 (111.68)	56.14 (33.32)	

Median (Q1, Q3)	36.30 (26.70, 51.20)	36.10 (26.10, 45.90)	56.00 (28.00, 75.80)	
Min, Max	15.00, 761.00	15.00, 761.00	18.00, 114.00	
**ALT (U/L)**				0.428
Mean (SD)	34.13 (33.82)	32.15 (32.36)	46.29 (42.57)	
Median (Q1, Q3)	22.95 (14.70, 40.30)	22.90 (15.00, 34.10)	43.10 (12.00, 75.00)	
Min, Max	7.10, 195.00	7.10, 195.00	8.00, 126.00	
**HBsAg positive**	1 (2.0%)	1 (2.3%)	0 (0.0%)	>0.999
**Anti-HCV positive**	3 (6.0%)	3 (7.0%)	0 (0.0%)	>0.999

[1] Continuous variables: mean (SD), median (IQR), range. Categorical variables: n (%). The small intermedia subgroup (n = 7) substantially limits the power of between-subtype comparisons; results are presented descriptively. Abbreviations: APTT, activated partial thromboplastin time; RBC, red blood cell; WBC, white blood cell; MCV, mean corpuscular volume; MCH, mean corpuscular hemoglobin; MCHC, mean corpuscular hemoglobin concentration; AST, aspartate aminotransferase; ALT, alanine aminotransferase; HBsAg, hepatitis B surface antigen; anti-HCV, hepatitis C antibody; TDT, transfusion-dependent thalassemia.

[2] Independent-samples t-test for continuous variables; Fisher’s exact test for categorical variables.

Figure S3. Distribution of key clinical and demographic variables by study group. Graphical comparison of continuous and categorical variables between patients with transfusion-dependent β-thalassemia (n = 50) and healthy controls (n = 30). Continuous variables are displayed as violin plots with embedded box plots; categorical variables are displayed as grouped bar charts. Groups are color-coded consistently throughout.

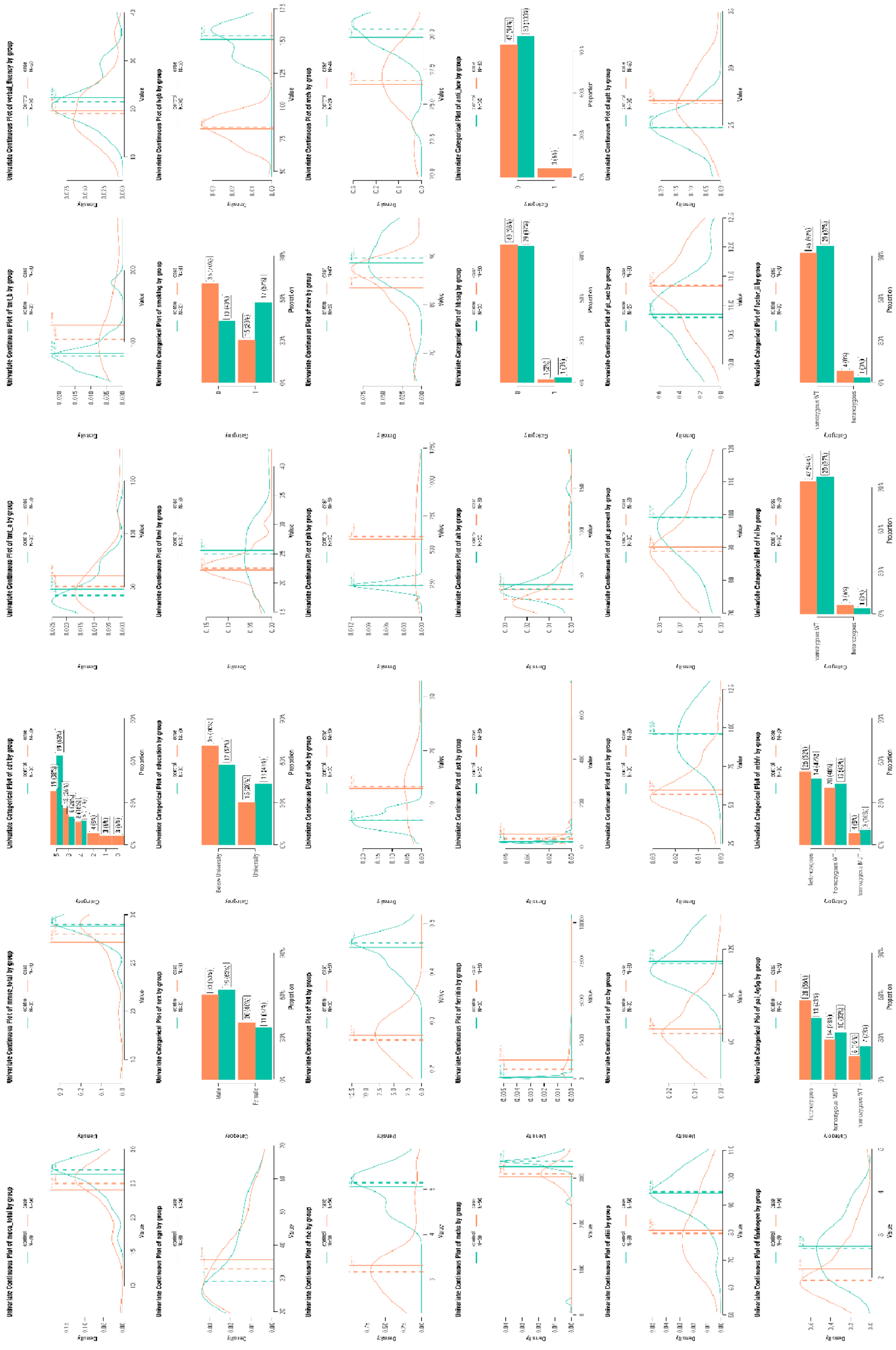

Supplement: Supplementary file 1 [file neurolint-18-00101-s001.zip › neurolint-4268677-supplementary.pdf]
